# Developing High-Resolution Descriptions of Urban Heat Islands: A Public Health Imperative

**DOI:** 10.5888/pcd13.160099

**Published:** 2016-09-15

**Authors:** Jackson Voelkel, Vivek Shandas, Brendon Haggerty

**Affiliations:** Author Affiliations: Jackson Voelkel, Nohad A. Toulan School of Urban Studies and Planning Portland State University, Portland, Oregon; Brendon Haggerty, Multnomah County Health Department, Portland, Oregon.

**Figure:**
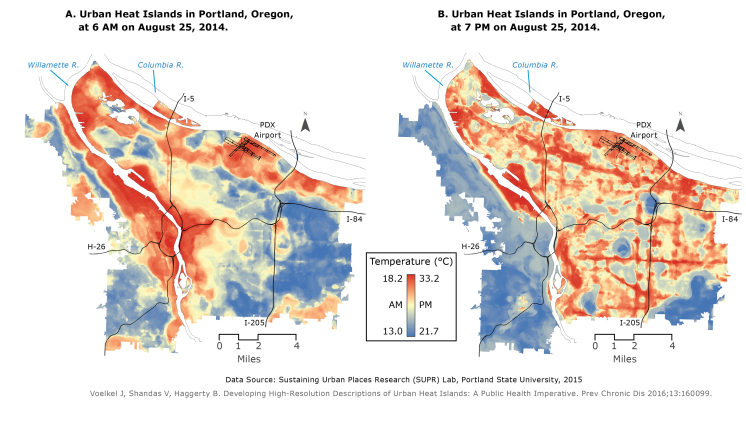
Empirically derived 1-m–resolution descriptions of (A) morning and (B) evening urban heat islands in Portland, Oregon, on August 25, 2014. Temperatures were recorded in 1-hour periods at 6 AM and 7 PM. In the morning, low-lying vegetation cover had the strongest effect on temperature; in the evening, temperatures were most strongly affected by variation in building heights. High-resolution data sets such as those used here can inform preparation for extreme heat events and public health interventions.

## Background

Extreme heat events affect the most vulnerable human populations and are a lethal health hazard to urban dwellers globally; in the United States, extreme heat causes more deaths annually than all other weather events and natural hazards combined (1). Previous studies described urban heat islands as isolated, static, monolithic areas of cities. We challenged this contention by hypothesizing that diurnal temperature cycles and diverse landscape features create variation in places that amplify heat (2). A temporal description of urban heat islands would identify populations that are susceptible to heat stress, particularly at night, when most people are asleep and unable to regulate internal body temperatures. If public health agencies are to prevent illness and death caused by heat, they will need to know which populations are most vulnerable to heat stress, particularly at night; such information can guide timely interventions (3). Researchers lack high-resolution tools for identifying neighborhoods and households where extreme weather events might have profound and fatal effects on human health. The objective of this study was to use spatial analytics at previously unattained resolutions to answer the following research question: to what extent can we observe temporal variation in urban heat islands and the physical features that induce heat stress?

## Methods

Following an established protocol (4), we collected approximately 60,000 temperature readings during 1 day of an extreme heat event on August 25, 2014, in Portland, Oregon, when the average temperature during the hottest hour of the day was in the 75th percentile of 30-year historic daily temperatures for the study region. We sampled temperatures for 1 hour at 3 times during the day (6 AM, 3 PM, and 7 PM) using vehicle traverses (cars with a mounted temperature sensor and global positioning system [GPS]) in 6 predetermined sections of the city. The temperature sensor consisted of a type T fine (30 gauge) thermocouple in a plastic shade tube (12 cm in length and 2.5 cm in diameter) mounted on the passenger-side window approximately 25 cm above the roof of each of 5 vehicles deployed. Each temperature sensor was connected to a data-logging device with an estimated system accuracy of ±0.5°C and a 90% response time of less than 60 s in 1 m/s airflow. A GPS unit on each vehicle paired temperature measurement and location.

On the basis of a sensitivity analysis and research on landscape features that mediate urban heat, we selected 6 variables as predictors: 1) building heights, 2) standard deviation of building heights, 3) building volume, 4) canopy cover, 5) low-lying vegetation, and 6) canopy biomass. Data on the first 3 variables were derived from 3-dimensional point cloud data acquired through Light Detection and Ranging (LiDAR). LiDAR combines a laser ranging device with a GPS system to provide highly resolved terrain measurements. The LiDAR data were extracted from the 2014 Oregon Metro Regional Land Information System (RLIS) (5). Data on canopy cover and low-lying vegetation were created from 2014 Portland LiDAR/photography flight data (5). The biomass metric was created by multiplying tree height by tree density determined by LiDAR-beam tree penetrability. Using a moving window analysis at 15 spatial extents (also known as “buffer distances”) from 50 m to 1 km, we tested the effective distances of each variable on the urban heat island, wherein each pixel represented the amount of each variable within a specified distance.

We analyzed modeling techniques (Appendix) and determined that random forest modeling (a machine-learning model available in the statistical package R [The R Foundation]) was more accurate than standard linear modeling. The values of the 90 new buffer-distance grids were spatially assigned to a randomly selected 70% of the traverse points; we tested the validity of our model by predicting the remaining 30%. This 70%–30% training model predicted a temperature for each of the 1.034 billion 1-m pixels. The models generated data on changes in mean standard error, which represents the effect of each on local temperature. Our statistical technique was run for each of the 3 one-hour data-collection periods, resulting in 3 temperature-prediction grids.

## Main Findings

The models for the morning and evening data-collection periods predicted upwards of 98% (*r* ^2^ = 0.98) and 97% (*r* ^2^ = 0.97) of the temperature variation across the study region; the afternoon model had a predicting power of 83% (*r* ^2^ = 0.83). Although the afternoon model was weaker, possibly because of atmospheric mixing and surface convective processes that we were unable to detect, it performed remarkably well.

In contrast with previous research findings, our models suggest that each data-collection period had unique land-use and land-cover factors that helped to explain variation (Table). In the morning (6 AM), low-lying vegetation cover had the strongest effect on temperature, and in the afternoon (3 PM), the 2 variables for building height (mean building heights and variation in building heights) had the strongest effect. In the evening (7 PM), temperatures were most strongly affected by the variation in building heights. Material science and computational fluid dynamics processes suggest that buildings absorb incoming solar radiation during the day and re-radiate it as heat at night (2) and that variation in building heights helps to circulate air. Indeed, diurnal patterns of extreme heat, documented in studies of heat mortality and urban heat islands, show that the re-radiation of heat by buildings peaks at night and places vulnerable individuals at greatest risk of death from heat. The amount of canopy cover may moderate temperatures at night because this variable is the strongest predictor in the morning.

**Table:** Landscape Factors and Their Relative Contribution to Urban Heat Islands at Three Times During One Day of an Extreme Heat Event in Portland, Oregon, August 2014

Model	Variable Rank^a^	Variable	Percentage Increase in Mean Standard Error	Model *r* ^2^	Model Mean Standard Error
6 AM	1	Low-lying vegetation cover within 50 m	42.5	0.98	0.02
	2	Low-lying vegetation cover within 800 m	38.7		
	3	Building volume within 900 m	33.9		
	4	Canopy biomass within 1000 m	33.0		
	5	Mean building height 100 m	32.7		

3 PM	1	Standard deviation of building height within 1000 m	40.8	0.83	0.23
	2	Standard deviation of building height within 300 m	44.8		
	3	Canopy biomass within 50 m	38.9		
	4	Standard deviation of building height within 150 m	38.7		
	5	Standard deviation of building height within 200 m	38.5		

7 PM	1	Standard deviation of building height within 1000 m	40.0	0.97	0.05
	2	Low-lying vegetation cover within 100 m	32.5		
	3	Building volume within 1000 m	30.9		
	4	Canopy cover within 800 m	30.9		
	5	Building volume within 900 m	30.6		

a Rank reflects the extent to which the variable explains the temperature throughout the study region.

## Action

By using these empirically derived heat measures, local land-use and land-cover variables, and spatial machine learning techniques, we described and explained variation in the distribution of urban heat islands in Portland, Oregon. High-resolution data sets and analysis such as those used here can inform preparation for extreme heat events and public health interventions (eg, information campaigns, cooling centers, tree planting programs, and surveillance) for vulnerable communities in local and regional areas. Our results also suggest that policy and environmental interventions should deploy temperature-mitigation strategies at night, when heat stress is greatest for vulnerable communities.

## Figures and Tables

**Figure:**
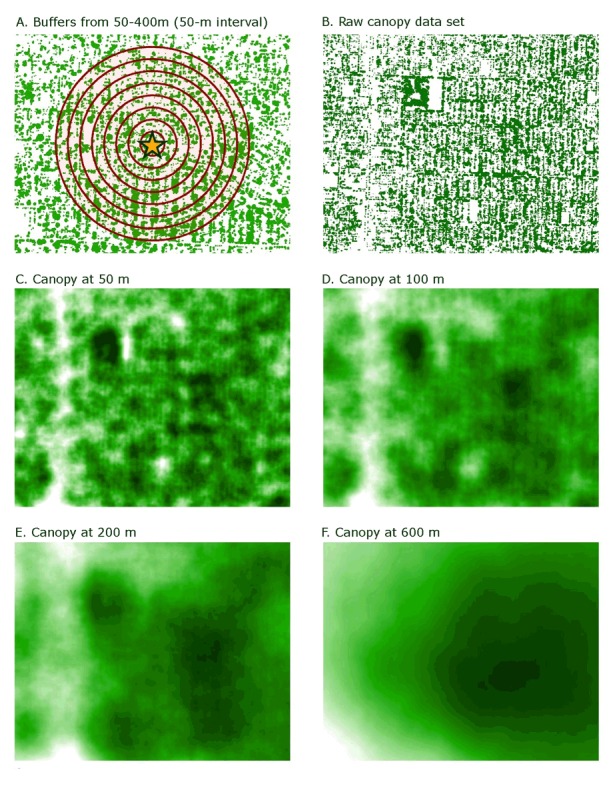
Example of the process of creating buffers on a raster. The input data (A) is converted from vector to a raster that contains pixel values representing the results for specified distances. In this example, the raw data has the values 1 (green, representing canopy) and 0 (white, representing not-canopy). The output describes the percentage of land cover classified as canopy.

## Appendix Additional Explanation of Analysis of Modeling Techniques to Determine That Random Forest Modeling Was More Accurate Than Standard Linear Modeling

We drew our buffer distances on the basis of studies that employ land-use regression (LUR) models. These studies describe similar decay rates for predicting the implication of land-use variables on environmental stressors (6). The literature on urban heat examines the relationship between land use and temperature using satellite-based approaches and attempts to correlate changes in temperature on a pixel-by-pixel approach (7). Because our techniques provide temporal characterization of urban heat through vehicle-based traverses on a single day, satellite-based approaches (which have a lower spatial and temporal resolution) are not a viable solution. Moreover, the existing literature on urban heat applies 1-km buffers as a maximum distance at which land-use variables have a significant effect on temperatures.

By transforming our raster data into a table in R Statistical Software (using the “raster” package), we are able to append each variable at each distance to a table with our observed temperature values. This table allows us to not only create a model, but to apply that model to our rasters to predict temperatures in areas where traverses did not collect data. We would be able to create the model by examining each temperature observation point and the land use within specified differences from them, however it would prove difficult to create an output model from that scenario. This method of raster-level analysis not only speeds up the process, but gives us much greater accuracy (no conversions between vector and raster) and the ability to create an output raster of our final prediction for analysis.

Based on the results of a sensitivity analysis comparing linear regression, classification and regression trees (CART), and random forest modeling, we found strongest explanatory power using random forest modeling (8).

The processes of creating buffers on a raster (also known as a moving window analysis or focal statistics) is a common practice of data manipulation in geographic information systems (Figure).
